# Placenta Praevia*Read before East Texas Medico-Chirurgical Society, Palestine, November 29, 1900.

**Published:** 1901-04

**Authors:** E. D. Stokes

**Affiliations:** Crockett, Texas


For Texas Medical Journal.

### Placenta Praevia.*

BY E. D. STOKES, M. D., CROCKETT, TEXAS.

Had it been left to the writer on this occasion to select a subject, a different one from this would have been chosen. The Program Committee, however, acted wisely in naming it as a topic for discussion.

I have looked through several hundred old journals and not an article could I find on Placenta Prævia. Have written my friends for literature, but all in vain. My writing will be largely extracts from text-books; don't think you would expect much more from one eight years in the practice and a very limited work.

Placenta Prævia consists in implantation of the placenta abnormally near to, or more or less over, the internal os uteri. Again, the placenta is said to be prævia when it occupies the lower uterine segment, that is, the portion of the uterus situated below the line of firm peritoneal attachment.

There are three varieties, viz.: marginal, partial and complete; the first approaching, the second slightly overlapping and the third completely overlapping the os.

The causes are various. Some of the most probable will follow: displacement of ovum; large relaxed uteri of multiparous women; abnormally low position of orifice of Fallopian tubes. Chronic endometritis, myomata and carcinomata are said to be predisposing causes. The absolute thuthfulness of any and all of these lacks confirmation.

The symptoms are not so varied, but they are shockingly suggestive when they do make their appearance. Hemorrhage is the main one, which begins without any known cause, sometimes at night during sleep, or while squatting down answering nature's call. It may stop and again recur.

The quantity varies with the extent of placental separation.

First attacks usually moderate, the quantity increasing with each recurrence. Even first attacks, however, do sometimes result in death.

If the gestation has reached full term (which is very uncommon) the bleeding begins with commencing dilatation of the os,

---

*Read before East Texas Medico-Chirurgical Society, Palestine, November 29, 1900.

which may be arrested (in marginal cases) by rupture of membranes. Labor pains are usually feeble and dilatation slow. To these symptoms must be added those due to blood-loss, syncope, restlessness, feeble pulse, cold extremities, vertigo, headache, etc. The diagnosis is confirmed by vaginal examination, the irregular, spongy texture of the placenta being recognized by the finger passed into the os. The stethoscope applied to cervix may reveal loud placental murmur.

Statistical estimates place maternal mortality at from 25 to 40%, but it is generally thought that this is too high. The prognosis is worse in proportion to the degree in which the placenta overlaps the os. Two-thirds of the infants are born dead and still others succumb shortly after birth.

The main principle of treatment is emptying the uterus. This should be done, regardless of whether the period of viability has been reached or not. The child will seldom be saved by temporizing and the mother often dies with the recurrence of the hemorrhage. But if even the sentimental issue alone is considered, there is little question but that a premature child has a better chance of survival than one the mother of whom has had repeated and copious losses of blood.

The usual method of delivery is by podalic version, preferably by the internal and external manipulation—the so-called "bipolar" method—and subsequent traction on feet, the leg, or the breech acting as a tampon when made to occupy the cervical canal. Some prefer to rely on the tampon of iodoform gauze, thoroughly packing the cervix and later the entire vagina. This must be well done. Under certain circumstances the gauze tampon is almost indispensable. For instance, when we have a cervix that is not dilated nor dilatable it is a right efficient means of controlling hemorrhage while we are waiting for sufficient dilation to admit of internal manipulation. This tampon should be removed after from two to four hours and vagina washed out with an antiseptic solution, after which version, formerly mentioned, may be carried out.

Insert two fingers into the os, locate the head, place other hand on abdomen, and while the hand inside shoves the head toward the iliac fossa, to which the occiput points, depress the breech with the hand outside, place fingers against shoulder and press head still higher, at the same time depress the breech more.

Usually by this time a knee can be reached, which is hooked and drawn down, the hand on the outside being used now to push head towards fundus.

After the foot is brought down the case is managed as an ordinary breech presentation, remembering that it is best, usually, to allow delivery to take place unaided.

Sometimes the placenta is in the way. If so, perforate the membranes at its margin, or at any point where it may be separated. Failing in this, plunge the fingers through the placenta, and bring down the leg through the opening thus made.

The maternal mortality is placed at four per cent. by some authorities if this method is skilfully carried out, and infant mortality sixty. Some successful practitoners completely separate that part of the placenta attached near the cervix, thus allowing retraction of the cervix, resulting in cessation of hemorrhage.

This procedure is said to be especially serviceable when the placenta is placed entirely over os. Frequently hemorrhage ceases and there is no necessity for further interference.

Closely resembling this last method (Barnes) is that of Cohen and Davis, which will not be given. Simpson's method of completely detaching placenta is admissable only when child is already dead, or can not live on account of prematurity, and when there is some strong contraindication to version. The use of ergot is not as objectionable as in other labors. Before using it, however, it should be ascertained that only the one mechanical obstruction exists.

Anemia, syncope, collapse, etc., from loss of blood will require stimulants. It is here that normal salt solution plays an important rôle. It may be given by rectum, injected into the tissues or directly into the blood vessel. After delivery ergot must be given for several days.

